# Supplementary material for: Fast alignment and preprocessing of chromatin profiles with Chromap
Source: Nat Commun. 2021 Nov 12;12:6566. doi: 10.1038/s41467-021-26865-w (PMC8589834; doi:10.1038/s41467-021-26865-w)

Supplementary materials for:

Fast alignment and preprocessing of chromatin profiles with Chromap

Haowen Zhang[1*], Li Song[2,3*], Xiaotao Wang[4], Haoyu Cheng[2,5], Chenfei Wang[2,3], Clifford A. Meyer[2,3,6], Tao Liu[7], Ming Tang[2], Srinivas Aluru[1,8], Feng Yue[4,9], X. Shirley Liu[2,3,6#], Heng Li[2,5#]

1: School of Computational Science and Engineering, Georgia Institute of Technology, Atlanta, GA, USA.
2: Department of Data Science, Dana-Farber Cancer Institute, Boston, MA, USA.
3: Harvard T.H. Chan School of Public Health, Boston, MA, USA
4. Department of Biochemistry and Molecular Genetics, Feinberg School of Medicine, Northwestern University, Chicago, IL, USA
5: Department of Biomedical Informatics, Harvard Medical School, Boston, MA, USA
6: Center for Functional Cancer Epigenetics, Dana-Farber Cancer Institute, Boston, MA, USA
7: Department of Biostatistics and Bioinformatics, Roswell Park Comprehensive Cancer Center, Buffalo, NY, USA
8: Institute for Data Engineering and Science, Georgia Institute of Technology, Atlanta, GA, USA
9: Robert H. Lurie Comprehensive Cancer Center of Northwestern University, Chicago, Illinois, USA
*: These authors contributed equally to this work.
#: Corresponding authors. Email: xsliu.res@gmail.com, hli@ds.dfci.harvard.edu

Supplementary Table 1. The statistics for the ChIP-seq, Hi-C and 10x Genomics scATAC-seq data sets.

	# of read pairs	Read length
ChIP-seq	37 million	101
Hi-C	1.4 billion	101
10x Genomics scATAC-seq	379 million	50

Supplementary Table 2. The computational cost of different methods on ChIP-Seq data. The preprocessing steps include MAPQ filtering, sorting and deduping.

	Time: alignment (min) + preprossing (min)	Memory (GB)
Chromap	3.5 + 1.5	18.4
Accel-Align	6 + 35	19.4
STAR	10 + 35	29.4
Minimap2	21 + 35	12.9
BWA-MEM	64 + 35	7.3
Bowtie2	86 + 35	3.5

Supplementary Table 3. The normalized mutual information (NMI) and adjusted rand index (ARI) of cell type annotations and cell clusters from MAESTRO on 10K PBMC 10x Genomics scATAC-seq data using Chromap and CellRanger v1.2.0 and v2.0.0. MAESTRO obtained 15, 16, 15 clusters from CellRanger v1.2.0, CellRanger v2.0.0 and Chromap results respectively.

Cluster			
	CellRanger_v1.2.0 vs CellRanger_v2.0.0	CellRanger_v1.2.0 vs Chromap	CellRanger_v2.0.0 vs Chromap
NMI	0.822	0.832	0.932
ARI	0.677	0.701	0.914
Cell type			
	CellRanger_v1.2.0 vs CellRanger_v2.0.0	CellRanger_v1.2.0 vs Chromap	CellRanger_v2.0.0 vs Chromap
NMI	0.922	0.933	0.964
ARI	0.963	0.969	0.983

Supplementary Table 4. The normalized mutual information (NMI) and adjusted rand index (ARI) of cell type annotations and cell clusters from ArchR on 10K PBMC 10x Genomics scATAC-seq data. ArchR obtained 11, 12, 13 clusters from CellRanger v1.2.0, CellRanger v2.0.0 and Chromap results respectively.

Cluster			
	CellRanger_v1.2.0 vs CellRanger_v2.0.0	CellRanger_v1.2.0 vs Chromap	CellRanger_v2.0.0 vs Chromap
NMI	0.865	0.881	0.899
ARI	0.896	0.905	0.928

Supplementary Table 5. The normalized mutual information (NMI) and adjusted rand index (ARI) of cell clusters from MAESTRO on 10K PBMC 10x Genomics scATAC-seq data using Chromap_bulkdedup and CellRanger v1.2.0 with BWA and Bowtie2 as aligners. MAESTRO obtained 15, 14, 15 clusters from BWA, Bowtie2 and Chromap results respectively.

Cluster			
	CellRanger_v1.2.0 vs CellRanger_v1.2.0_Bt2	CellRanger_v1.2.0 vs Chromap_bulkdedup	CellRanger_v1.2.0_Bt2 vs Chromap_bulkdedup
NMI	0.903	0.918	0.927
ARI	0.884	0.919	0.916

Supplementary Figure 1. Intersections of peaks called from Accel-Align, STAR and minimap2 alignments respectively with the peaks called from BWA-MEM and Bowtie2 alignments on bulk ChIP-seq data.

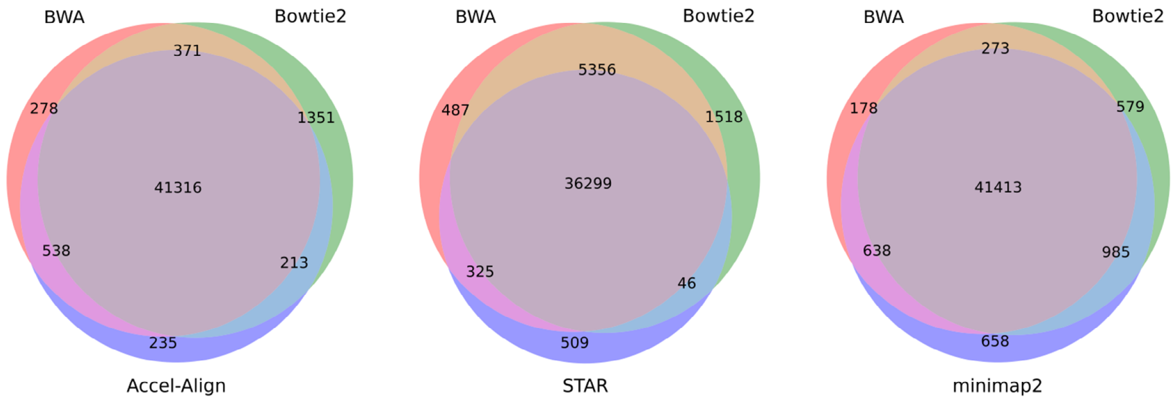

Supplementary Figure 2. Annotations of peaks called by MACS2 using BWA-MEM, Bowtie2, minimap2, STAR, Accel-Align and Chromap alignments on bulk ChIP-seq data.

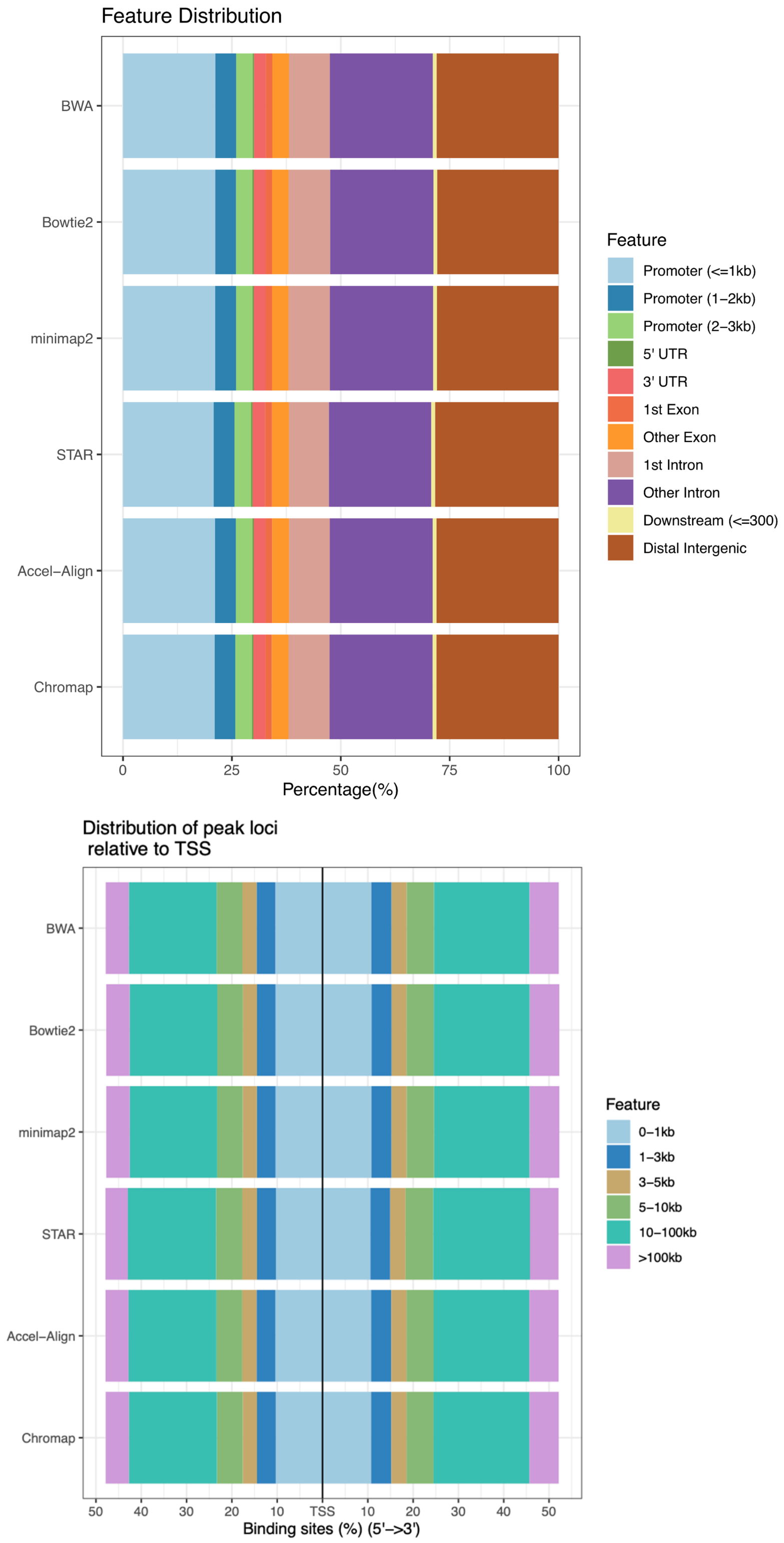

Supplementary Figure 3. The number of overlapped peaks generated using MACS2 on BWA-MEM, Bowtie2, Chromap alignments of ChIP-seq replicate 1 and on BWA-MEM alignments of ChIP-seq replicate 2 (denoted as BWA (replicate)).

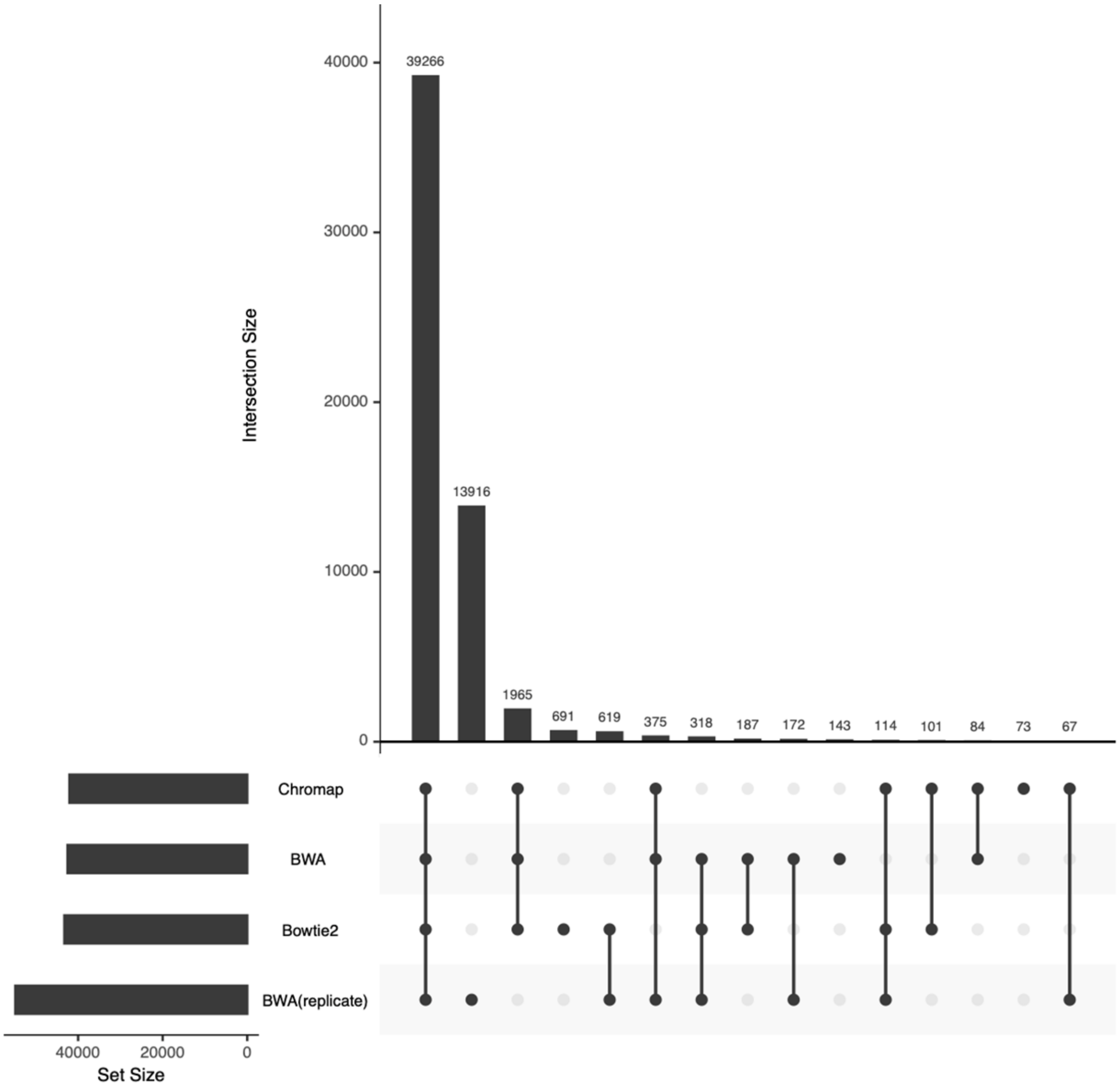

Supplementary Figure 4. Evaluation of the Hi-C data

(a) Contact matrices at 100kb resolution and compartment consistency based on Chromap and BWA-MEM.

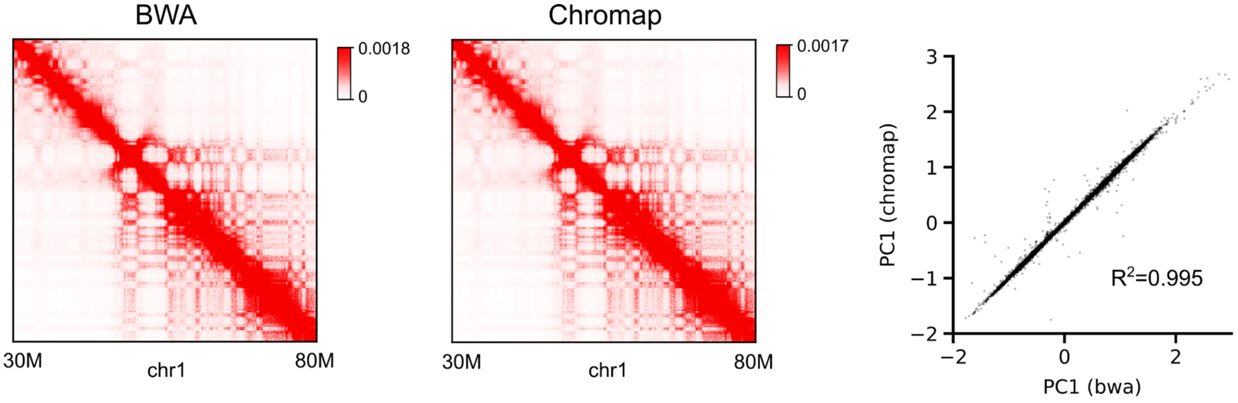

(b) Chromatin loops based on Chromap and BWA-MEM

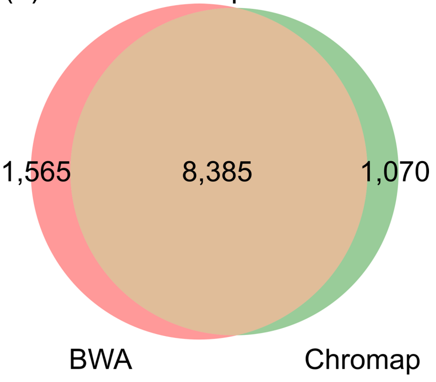

(c) Average CTCF supports around Chromap-unique and BWA-unique loop anchor site

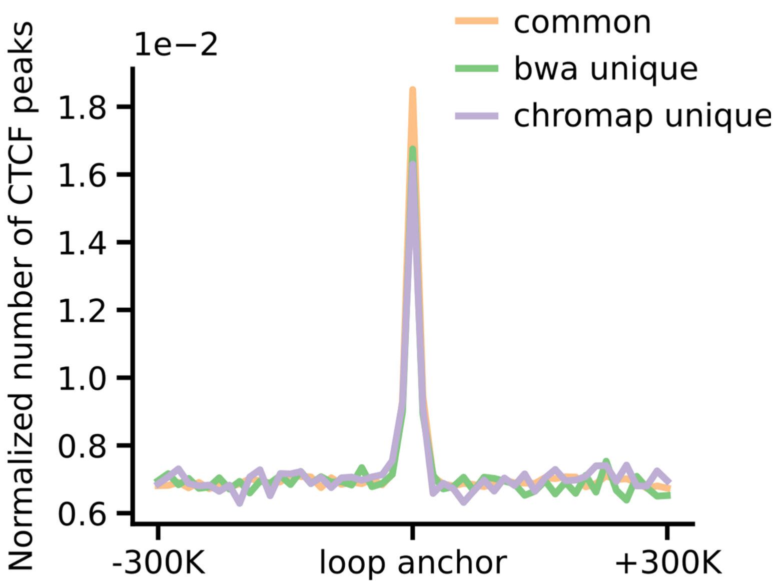

Supplementary Figure 5. An example of adapter removal. This read pair has forward R1 TTGACTGGACACGA and backward R2 GTCCAGGCAATCGT (reverse-complement ACGATTGCCTGGAC denoted as $R2^T$). Suffix of $R2^T$ can be matched to the prefix of R1 with an overlap size of 10 including 1 mismatch, indicating fragment length is shorter than read length. Therefore, Chromap removes ACGA from R1 and $R2^T$ (TCGT in R2) as parts of the adapter sequences.

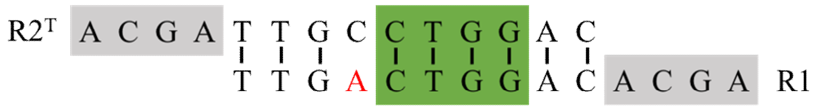

Supplement: Supplementary file 1 — Supplementary Information [file 41467_2021_26865_MOESM1_ESM.pdf]
