# Supplementary material for: Fast alignment and preprocessing of chromatin profiles with Chromap
Source: Nat Commun. 2021 Nov 12;12:6566. doi: 10.1038/s41467-021-26865-w (PMC8589834; doi:10.1038/s41467-021-26865-w)
Supplement: Supplementary file 2 — Reporting Summary [file 41467_2021_26865_MOESM2_ESM.pdf]

nature portfolio

Corresponding author(s): X. Shirley Liu; Heng Li

Last updated by author(s): Oct 8, 2021

Reporting Summary

Nature Portfolio wishes to improve the reproducibility of the work that we publish. This form provides structure for consistency and transparency in reporting. For further information on Nature Portfolio policies, see our Editorial Policies and the Editorial Policy Checklist.

Statistics

For all statistical analyses, confirm that the following items are present in the figure legend, table legend, main text, or Methods section.

n/a	Confirmed	
☐	☒	The exact sample size (*n*) for each experimental group/condition, given as a discrete number and unit of measurement
☐	☒	A statement on whether measurements were taken from distinct samples or whether the same sample was measured repeatedly
☒	☐	The statistical test(s) used AND whether they are one- or two-sided<br>*Only common tests should be described solely by name; describe more complex techniques in the Methods section.*
☒	☐	A description of all covariates tested
☒	☐	A description of any assumptions or corrections, such as tests of normality and adjustment for multiple comparisons
☒	☐	A full description of the statistical parameters including central tendency (e.g. means) or other basic estimates (e.g. regression coefficient) AND variation (e.g. standard deviation) or associated estimates of uncertainty (e.g. confidence intervals)
☒	☐	For null hypothesis testing, the test statistic (e.g. *F*, *t*, *r*) with confidence intervals, effect sizes, degrees of freedom and *P* value noted<br>*Give P values as exact values whenever suitable.*
☒	☐	For Bayesian analysis, information on the choice of priors and Markov chain Monte Carlo settings
☒	☐	For hierarchical and complex designs, identification of the appropriate level for tests and full reporting of outcomes
☐	☒	Estimates of effect sizes (e.g. Cohen's *d*, Pearson's *r*), indicating how they were calculated

Our web collection on statistics for biologists contains articles on many of the points above.

Software and code

Policy information about availability of computer code

Data collection	ChIP-seq data with two replicates are available from ENCODE: ENCSR265ARE<br>10x Genomics data is available at: https://support.10xgenomics.com/single-cell-atac/datasets/1.2.0/atac_v1_pbmc_10k<br>Hi-C data is available in the SRA repositories SRR1658693~SRR1658702.
Data analysis	We used BWA v0.7.17, Bowtie2 v2.4.2, minimap2 v2.17, STAR v2.7.9a, Accel-align github 7217a9f, Mason v2.0.9, samtools v1.10, Picard v2.23.6, ChIPSeeker v1.24.0, pairtools 0.3.0, HiCRep v0.0.6, cooltools v0.3.2, HiCCUPS v0.3.4, CellRanger v1.2.0, CellRanger v2.0.0, MAESTRO v1.4.0, ArchR v1.0.1 , Chromap v0.1.1 .<br><br>The code used in the evaluation is available at: https://github.com/haowenz/chromap_evaluation<br>Chromap source code is available at https://github.com/haowenz/chromap.

For manuscripts utilizing custom algorithms or software that are central to the research but not yet described in published literature, software must be made available to editors and reviewers. We strongly encourage code deposition in a community repository (e.g. GitHub). See the Nature Portfolio guidelines for submitting code & software for further information.

Data

Policy information about availability of data

All manuscripts must include a data availability statement. This statement should provide the following information, where applicable:

- Accession codes, unique identifiers, or web links for publicly available datasets
- A description of any restrictions on data availability
- For clinical datasets or third party data, please ensure that the statement adheres to our policy

ChIP-seq data with two replicates are available from ENCODE: ENCSR265ARE
10x Genomics data is available at: https://support.10xgenomics.com/single-cell-atac/datasets/1.2.0/atac_v1_pbmc_10k
Hi-C data is available in the SRA repositories SRR1658693~SRR1658702.

Field-specific reporting

Please select the one below that is the best fit for your research. If you are not sure, read the appropriate sections before making your selection.

☒ Life sciences ☐ Behavioural & social sciences ☐ Ecological, evolutionary & environmental sciences

For a reference copy of the document with all sections, see nature.com/documents/nr-reporting-summary-flat.pdf

Life sciences study design

All studies must disclose on these points even when the disclosure is negative.

Sample size	We generated three simulated data, downloaded one ChIP-seq sample from ENCODE with two replicates, one Hi-C sample with two replicates, and one 10x Genomics single-cell ATAC-seq data. We select three simulated data for three different read lengths. Each scenario has one sample to measure the performance of the computational methods. Since we are not conducting statistical comparisons, there is no need to have multiple samples.
Data exclusions	No data were excluded from the analysis.
Replication	We have replicates in ChIP-seq and Hi-C data to show the difference introduced by alternating computational methods is less than biological replicates. It is not for reproduce biological findings.
Randomization	Not relevant because we only compared the performance of computational methods and did not draw biological conclusions.
Blinding	Not relevant because we only compared the performance of computational methods and did not draw biological conclusions.

Reporting for specific materials, systems and methods

We require information from authors about some types of materials, experimental systems and methods used in many studies. Here, indicate whether each material, system or method listed is relevant to your study. If you are not sure if a list item applies to your research, read the appropriate section before selecting a response.

Materials & experimental systems

n/a	Involved in the study	
☒	☐	Antibodies
☒	☐	Eukaryotic cell lines
☒	☐	Palaeontology and archaeology
☒	☐	Animals and other organisms
☒	☐	Human research participants
☒	☐	Clinical data
☒	☐	Dual use research of concern

Methods

n/a	Involved in the study	
☒	☐	ChIP-seq
☒	☐	Flow cytometry
☒	☐	MRI-based neuroimaging
